# Supplementary material for: Narrowing the A1c gap: Personalized modeling of HbA1c– continuous glucose monitor discordance in type 1 diabetes
Source: PLOS Digit Health. 2026 Feb 17;5(2):e0001229. doi: 10.1371/journal.pdig.0001229 (PMC12912621; doi:10.1371/journal.pdig.0001229)
Supplement: S1 Text — (DOCX) [file pdig.0001229.s001.docx]

**Discordance prevalence**

**Cohort A:** An absolute discordance of ≥0.5% and ≥1% between paired measurements were observed in 34% and 8% of cases, respectively. In 51% of instances, the direction of discordance (i.e., whether the value was higher or lower) was consistent across consecutive measurement pairs for the same individual.

**Cohort B:** An absolute discordance of ≥0.5% and ≥1% between paired measurements were observed in 25% and 4% of cases, respectively. Similar to Cohort A, 51% of cases exhibited a consistent direction of discordance across consecutive measurement pairs within individuals.
