# Supplementary material for: Narrowing the A1c gap: Personalized modeling of HbA1c– continuous glucose monitor discordance in type 1 diabetes
Source: PLOS Digit Health. 2026 Feb 17;5(2):e0001229. doi: 10.1371/journal.pdig.0001229 (PMC12912621; doi:10.1371/journal.pdig.0001229)
Supplement: S1 Fig — Sankey plots show the progression from the baseline discordance groups (left) – positive and negative – over time to measurement 1 (center) and measurement 2 (right). (DOCX) [file pdig.0001229.s002.docx]

**Cohort A (Sankey)**

**
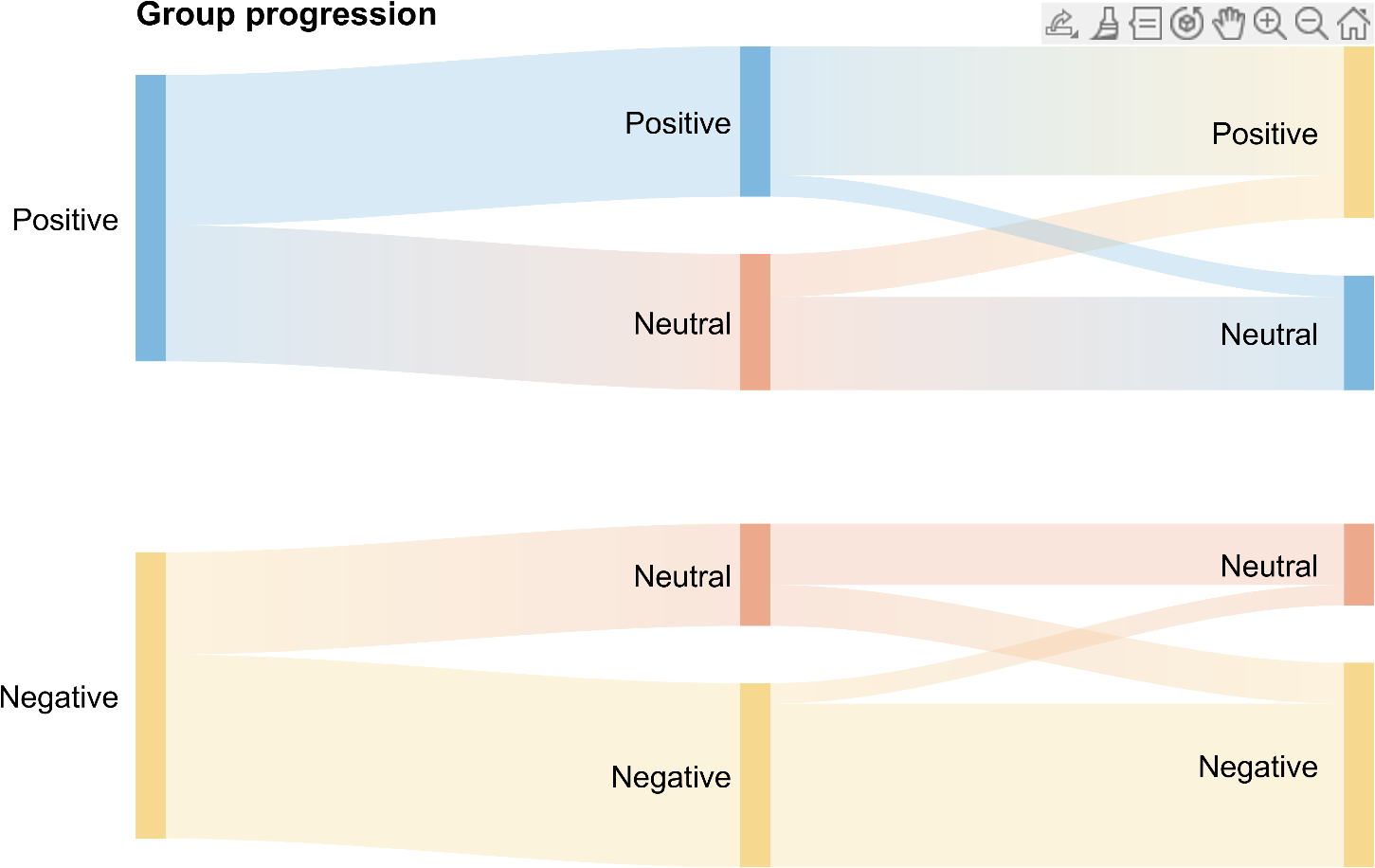
**

**Figure S1 – Discordance group progression over time (Cohort A / The T1DiabetesGranada study).**Sankey plots show the progression from the baseline discordance groups (left) – positive and negative – over time to measurement 1 (center) and measurement 2 (right).
