# Supplementary material for: Narrowing the A1c gap: Personalized modeling of HbA1c– continuous glucose monitor discordance in type 1 diabetes
Source: PLOS Digit Health. 2026 Feb 17;5(2):e0001229. doi: 10.1371/journal.pdig.0001229 (PMC12912621; doi:10.1371/journal.pdig.0001229)
Supplement: S4 Fig — The development in discordance in the groups – positive (discordance ≥0.5), neutral (-0.5 > discordance<0.5) and negative (-0.5≤) - from baseline to the first and second measurement. (DOCX) [file pdig.0001229.s005.docx]

**Cohort B (Boxplot)**


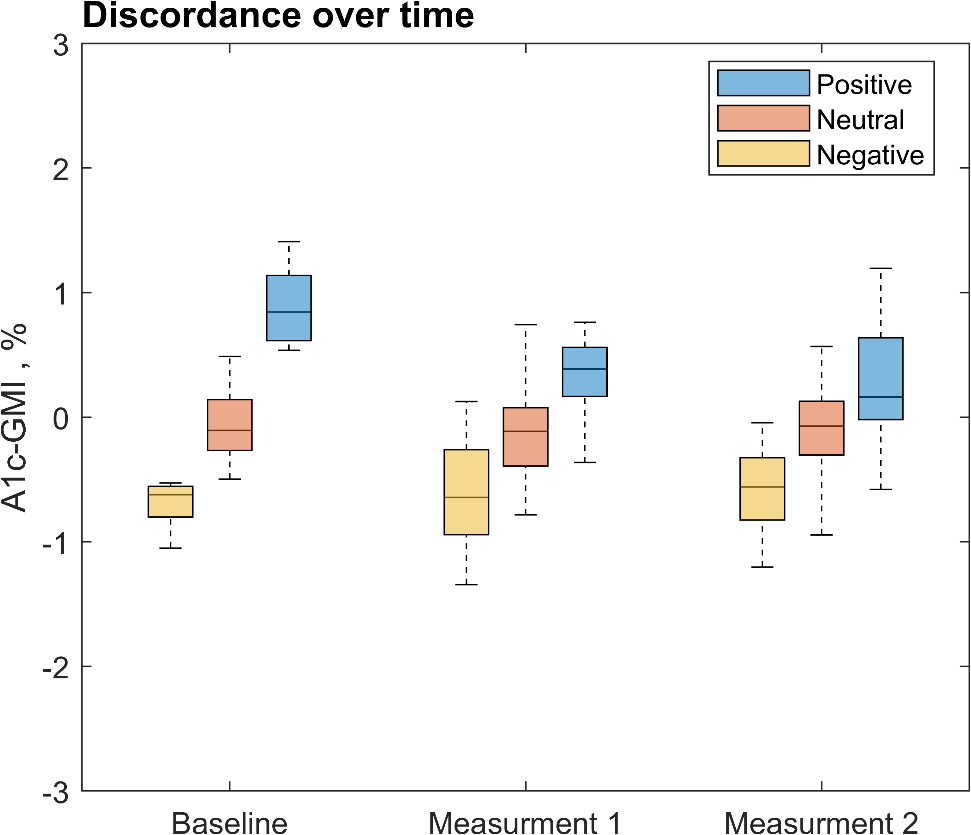


**Figure S4 – Discordance groups over time (Cohort B / The REPLACE-BG trial).**The development in discordance in the groups – positive (discordance ≥0.5), neutral (-0.5>discordance<0.5) and negative (-0.5≤) - from baseline to the first and second measurement.
